# Come Together: Protein Assemblies, Aggregates and the Sarcostat at the Heart of Cardiac Myocyte Homeostasis

**DOI:** 10.3389/fphys.2020.00586

**Published:** 2020-06-04

**Authors:** Moydul Islam, Abhinav Diwan, Kartik Mani

**Affiliations:** ^1^Division of Cardiology, Washington University School of Medicine, St. Louis, MO, United States; ^2^Center for Cardiovascular Research, Washington University School of Medicine, St. Louis, MO, United States; ^3^Department of Chemistry, Washington University in St. Louis, St. Louis, MO, United States; ^4^John Cochran Veterans Affairs Medical Center, St. Louis, MO, United States

**Keywords:** aggregates, autophagy, ubiquitin 26S-proteasome system, lysosome, heat shock proteins

## Abstract

Homeostasis in vertebrate systems is contingent on normal cardiac function. This, in turn, depends on intricate protein-based cellular machinery, both for contractile function, as well as, durability of cardiac myocytes. The cardiac small heat shock protein (csHsp) chaperone system, highlighted by αB-crystallin (CRYAB), a small heat shock protein (sHsp) that forms ∼3–5% of total cardiac mass, plays critical roles in maintaining proteostatic function via formation of self-assembled multimeric chaperones. In this work, we review these ancient proteins, from the evolutionarily preserved role of homologs in protists, fungi and invertebrate systems, as well as, the role of sHsps and chaperones in maintaining cardiac myocyte structure and function. We propose the concept of the “sarcostat” as a protein quality control mechanism in the sarcomere. The roles of the proteasomal and lysosomal proteostatic network, as well as, the roles of the aggresome, self-assembling protein complexes and protein aggregation are discussed in the context of cardiac myocyte homeostasis. Finally, we will review the potential for targeting the csHsp system as a novel therapeutic approach to prevent and treat cardiomyopathy and heart failure.

## Introduction

Evolving over the previous 600 million years (Poelmann and Gittenberger-de Groot, 2019), the metazoan circulatory system is at the center of the explosion of multicellular functionality, culminating in the human era. From primordial heart tubes in early protostomes to the four-chambered hearts of mammals, each of these circulations have ensured nutrient and oxygen supply, maintenance of temperature, as well as waste removal. In each instance, the circulatory system is driven by contractile cells, from the endo-symbiotic contractile elements in ancient protists a billion years ago, to the cardiac myocytes of chordates. The highlight of all these cells is a complex network of protein assemblies that form organized contractile machines. Cardiac myocytes are unique in lifespan, size, structure, function, durability, and metabolism. Each of these features is essential in ensuring continuous uninterrupted cardiac function, from early embryo, through the entire duration of post-natal life. Morphologically, the cardiac syncytium is similar to that observed in many protists and yeasts. In addition to the unusually large energetic apparatus, essential for powering the contractile machinery, cardiac myocytes, like yeast, require a complex proteostatic system. Unlike the dominant role of the proteasome in other cell types, cardiac myocyte lysosome function, like the vacuole in bacteria, plants and yeast, plays a major role in integrating metabolism (Mani et al., 2018) with both synthetic and degradation machinery for the contractile proteins. Thus, lysosome dysfunction results in metabolic derangement, as well as, proteotoxicity. This ultimately presents as cardiomyopathy and heart failure (Platt et al., 2012). Given the enormous and dynamic proteostatic load, in both long-lived monads as well as cardiac myocytes, evolutionarily preserved chaperones, like the small heat shock protein, αB-crystallin (CRYAB), play a critical role in maintaining cardiac homeostasis. sHsp mutations as well as those in proteostatic machinery components, such as BAG3, have been implicated as mediators of cardiomyopathy. In this review, we highlight the role of these proteostatic systems that constitute the underpinnings on normal cardiac function, as well as their roles, both, as arbiters of heart failure and, as potential novel therapeutic targets.

## Proteostatic Failure in Heart Disease: *The Devil in the Heart*

Given the primacy of their contractile function in homeostasis, diseases affecting cardiac myocytes result in impairment of, either or both, systolic (contractile) and diastolic (relaxation) properties of the heart. This ultimately presents as cardiomyopathy and heart failure leading to death. From a public health point of view, unlike advances in infectious diseases and cancer, epidemiologic indices have lagged with regards to the societal impact of cardiovascular disease, especially heart failure (Virani et al., 2020). This is despite considerable advances in neuro-hormonally targeted approaches in pharmacotherapy, as well as hemodynamically focused and perfusion-directed invasive procedures and devices. A critical lacuna remains in development of strategies specifically directed toward the cardiac contractile system. Notably, with the limited efforts to augment or replace cardiac contractility, current approaches in the form of mechanical assist devices, stem cell therapy and, select pharmacological therapies, have minimal overall lasting benefits. Furthermore, recent epidemiologic trends show a worrisome reversal of hard-fought prior gains with improvements in cardiovascular mortality and mortality (Virani et al., 2020), dictating a sense of urgency. Optimal cardiac function depends on both optimal function of the cardiac myocytes as well as preservation of the tissue architecture. Thus, targeting the protein quality control systems in rebuilding, and possibly enhancing, the unique cardiac contractile protein network, *in situ*, is a very attractive strategy.

Cardiomyopathies are traditionally classified by etiology, either as sequelae of ischemic heart disease (due to occlusion of coronary arteries) [ischemic cardiomyopathy (ICM], or those without ischemic insults [non-ischemic cardiomyopathy (NICM)] (McKenna et al., 2017). Despite this differentiation, the transition from incipient to overt heart failure in both forms of cardiomyopathy shares many common mechanisms such as incremental hemodynamic stresses, metabolic alterations, abnormal tissue perfusion and inflammatory changes. Abnormalities in protein quality control also appear to play roles in both ischemic and non-ischemic dilated cardiomyopathy (reviewed in Henning and Brundel, 2017). Similar to the “second hit” hypothesis in malignant transformation, it has been suggested that genetic predisposition in the form of abnormal protein quality control may provoke changes in the sarcomere that result in cardiomyopathy and heart failure (Predmore et al., 2010; Herrmann et al., 2013; Rainer et al., 2018). Indeed, while mutations in candidate protein quality control machinery proteins like αB-crystallin, BAG3, HspB7, Vps34, p97 appear to play mechanistic roles in inducing dilated cardiomyopathy in genetic myofibrillar myopathies (Vicart et al., 1998; Bova et al., 1999; Fang et al., 2017; Judge et al., 2017; Kimura et al., 2017; Dominguez et al., 2018; Brody et al., 2019); variants in many of these, such as BAG3 and KLHL3 are also associated with cardiomyopathy in population-based genomic analyses (Aragam et al., 2018; Myers et al., 2018; Shah et al., 2020). Conversely, mutations in sarcomeric components like desmin, titin, actin, myosin, myosin binding protein C (cMyBP-C) are associated with features of abnormal proteostasis and are similarly reflected in population genomic studies as well (Goldfarb and Dalakas, 2009; Herman et al., 2012; Schlossarek et al., 2012; Esslinger et al., 2017; Hoorntje et al., 2017; McNally and Mestroni, 2017; Glazier et al., 2018). Interestingly, in humans, autosomal recessive deletion-mutant of αB-crystallin at *M1* (Ma K. et al., 2019) as well as missense mutations in this protein at *D109A* (Fichna et al., 2017), *D109H* (Sacconi et al., 2012), *R120G* (Vicart et al., 1998), and *R157H* (Inagaki et al., 2006) result in early fatality. In contrast, a murine model of αB-crystallin ablation (which is accompanied with unintended ablation of Hspb2; Brady et al., 2001) results primarily in skeletal myopathy which manifests with aging; and forced overexpression of αB-crystallin mutants (such as R120G) has been required to replicate human cardiomyopathy phenotype in a much more insidious and delayed fashion (Wang et al., 2001; Rajasekaran et al., 2007). This highlights differences in protein quality control mechanisms between species, whereby caution is warranted in interpreting findings from murine experiments as models of human disease.

A key feature of protein quality control pathways is the efficient functioning of systems to degrade misfolded, damaged and potentially superfluous proteins. Tagging of proteins to target them for degradation is typically achieved by ubiquitination, which involves a covalent attachment of the protein to a 76 amino acid protein ubiquitin moiety to a lysine residue; which may further expand via a variety of branching mechanisms to achieve poly-ubiquitination (Kwon and Ciechanover, 2017). In the process of poly-ubiquitination, a ubiquitin moiety can be conjugated to another one via one of its seven lysine residues or its methionine residue to confer specificity in further processing of the host protein that is ubiquitinated. Ubiquitination of proteins can confer signaling roles, or result in degradation of the protein. Studies have demonstrated that linkages via lysine 48 (K48) and lysine 11 (K11) target proteins for degradation via the proteasome, whereas lysine 63 (K63) linkages confer signaling roles or are targeted for degradation via the autophagy-lysosome pathway (Kwon and Ciechanover, 2017). The proteasome is a specialized organelle in the cell comprised of a complex of proteolytic enzymes organized in two subunits, a catalytic 20S subunit; and a regulatory 19S subunit, which together form a cylindrical structure that de-ubiquitinates, unfolds and cleaves peptide bonds in proteins to generate amino acids (Bard et al., 2018). Ubiquitin tagging of aggregate-prone proteins is essential for their efficient removal (Galves et al., 2019). However, studies have documented impairment in the ubiquitin proteasome pathways by mutant αB-crystallin (R120G) (Chen et al., 2005; Zhang et al., 2010, 2019; Gupta et al., 2014) and desmin mutants (Liu et al., 2006a, b) that are linked to cardiomyopathy in humans; suggesting that worsening protein aggregate pathology is linked at least in part to progressive impairment in this arm of the protein quality control machinery. Moreover, recent studies conclusively demonstrate rapid development of fulminant cardiomyopathy and death in mice lacking *Psmc1* (that encodes for an essential component of the 19S proteasome subunit) (Pan et al., 2020); with concomitant upregulation of the autophagy-lysosome machinery as an adaptive response to remove accumulated protein aggregates.

Autophagy, a lysosomal degradative pathway that sequesters proteins, organelles and other cellular constituents is another mechanism for degradation of long-lived proteins and damaged proteins, and acts as a back-up in the setting of proteasome dysfunction (Pohl and Dikic, 2019). Autophagy or “self-eating” occurs via multiple pathways that involve sequestration of cargo within double membrane bound autophagosomes (termed as macroautophagy), direct uptake of proteins with a specific KFERQ motif into the lysosome via chaperone-mediated autophagy, or lysosomal membrane invagination and sequestration of proteins via microautophagy (reviewed in Dikic and Elazar, 2018). Studies from our lab as well as others have demonstrated that the autophagy-lysosome pathway also becomes progressively impaired with expression of aggregate-prone proteins in cardiac myocytes, such as R120G mutant of αB-crystallin (Ma X. et al., 2019; Pan et al., 2019); which is mechanistically secondary to suppression of the lysosome biogenesis program. Conversely, many lysosomal disorders including Danon’ disease, Pompe’s disease and Fabry’s disease result in a cardiomyopathy with evidence of failed proteostasis (reviewed in Sciarretta et al., 2018a). Failure of the ubiquitin-proteasome system and autophagy-lysosome pathways has also been implicated in more common forms of cardiomyopathy and heart failure resulting from ischemia-reperfusion injury and pressure overload stress (reviewed in Wang et al., 2011). Specifically, we have uncovered evidence for lysosome impairment in cardiac myocytes (Ma et al., 2012a, b) and macrophages (Javaheri et al., 2019) in the setting of myocardial ischemia-reperfusion injury, at least in part due to suppression of the lysosome biogenesis program (Godar et al., 2015; Ma et al., 2015). This impairment of autophagy-lysosome pathway is associated with accumulation of poly-ubiquitinated proteins (Godar et al., 2015), pointing to a critical role for this pathway in protein quality control in the setting of ischemia-reperfusion injury. Moreover, autophagy suppression is also observed in the chronic phase after myocardial infarction and contributes to development of ischemic cardiomyopathy (Maejima et al., 2013). This is associated with formation of “aggresomes” which are p62-containing protein aggregates formed as a cellular response to sequester mis-folded and damaged proteins when their removal is impeded (Johnston et al., 1998). In this study (Maejima et al., 2013), the autophagy suppression was mechanistically driven by activation of Mst1 (mammalian Ste20-like kinase 1), a serine-threonine kinase component of the Hippo signaling pathway, which is sufficient to phosphorylate Beclin-1 to promote its sequestration by Bcl-2 and inhibit autophagosome formation. Indeed, work from this group subsequently demonstrated that stimulation of autophagy-lysosome pathway with trehalose was effective in clearing p62 and rescuing post-myocardial infarction ventricular dilation and dysfunction (Sciarretta et al., 2018b). p62 has also been described to play a critical role in aggresome formation in the setting of R120G αB-crystallin mutant or desmin mutant expression, which protects cardiac myocytes from cell death (Zheng et al., 2011). Other components of the aggresome have been uncovered in studies with laser microdissection of intracytoplasmic inclusions identified in muscle biopsies from patients with reducing body myopathy (RBM) which led to the identification of mutations in Xq26.3-encoded four and a half LIM domain 1 (FHL1) protein as a cause for cardiomyopathy (Schessl et al., 2008).

Impairment of the autophagy-lysosome pathway has also been described with progression of pressure-overload induced hypertrophy and cardiac failure (reviewed in Sciarretta et al., 2018a), which accompanies failure of ubiquitin proteasome system and impaired protein quality control (Wang et al., 2011). Recent also studies demonstrate that coupling of poly-ubiquitinated proteins to extra-proteasomal receptors, specifically Ubiquilin 1, plays an important role in removal of K48-linked poly-ubiquitinated substrates in cardiac myocytes to maintain homeostasis in response to ischemia-reperfusion stress (Hu et al., 2018). Conversely, activation of the ubiquitin-proteasome system or the SUMOylation pathways (with UBC9 overexpression) were sufficient to rescue many features of cardiomyopathy induced by expression of the R120G αB-crystallin mutant (Gupta et al., 2016). Taken together, these observations suggest an intricate relationship in the delicate balance between the sarcomere and proteostatic systems that are key to the otherwise robust, durable and reliable functioning of the sarcomere and the cardiac myocyte. Conceivably, while inciting event may differ based upon the individual pathology, failed proteostasis machinery and increased abundance of aggregate-prone protein may be part of a vicious cycle where either impairments trigger a feed forward loop to drive the pathology.

## Sarcomeres: *Something in the Way They Move*

**FIGURE 1:**
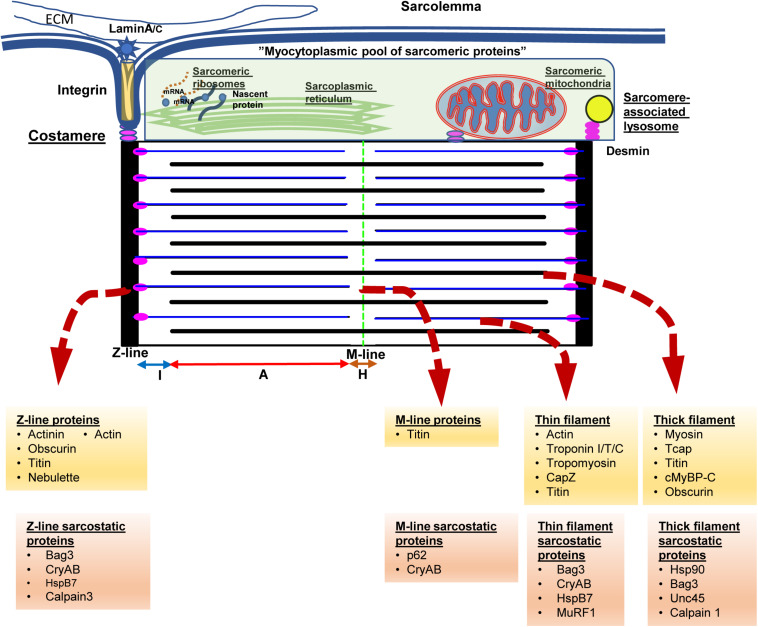
The sarcomere from the protein quality control perspective. Cardiac myocytes contain multiple myofilament bundles running through the length of the cell and are attached on either end to the intercalated disk between adjacent cells. Each myofilament, in turn, consists of a series of repeating units of contractile assemblies, known as sarcomeres. Each sarcomere is 2.2 microns in length and extends between two electron dense lines, the Z-lines with alternating areas of isotropic (I band) and anisotropic (A band) with a slender electron dense central M-line. The A band consists of an overlap zone with both thick and thin filaments, while the I band extends from the Z-line consisting of only thin filaments. Around the M-line, there is a relatively isotropic area consisting of only thick filaments known as the H-zone. The thin filaments are attached to the Z-lines via the anchor protein desmin, which also results in anchoring sarcomeric mitochondria and lysosomes to the sarcomere. Each sarcomere is also attached via integrins and laminins to the extracellular matrix via costameres that also anchor the sarcomere to the sarcolemma as well as to the intercalated disk. Disruption of desmin results in disruption of the sarcomere, mitochondrial dysfunction, and cardiac myocyte dysfunction, ultimately presenting with fatal cardiomyopathy. The Z-lines are formed via assemblies of actinin, actin, as well as a host of Z-line associated proteins. The underlying skeleton of the entire sarcomere is created via the giant proteins, titn, obscurin, and nebulette. The titin sarcomeric skeleton extends from the Z-line to the M-line. Over the titin skeleton, thick, and thin filaments are assembled. High energy phosphate driven movement of the myosin (thick filaments) against the actin filaments, triggered by calcium fluxes from sarcolemmal depolarization, results in shortening of the sarcomere. Summation of sarcomere contraction results in cardiac myocyte and ultimately, the heartbeat. Cognate chaperones (e.g., unc-45) as well as multifunctional chaperones [CRYAB (αB-crystallin)/Bag3] play roles in maintaining the sarcomere and are listed above. The proteins at the above noted structures and associated chaperones are listed underneath in yellow and orange background.

In order to understand the key function of cardiac myocytes, it is imperative to focus on the complex protein architecture that define the essential contractile unit of the heart, namely the sarcomeres (see Figure 1). Consisting of repeating units of a near-identical arrangement of contractile, non-contractile proteins as well as a complex of regulatory proteins, up to 300 of these 2.2 μm-long sarcomeres are joined end-to-end to form the myofibrils that power cardiac contractility (Clark et al., 2002; van der Velden and Stienen, 2019; see Figure 1). Thus, each myofibril consists of repeating sarcomeres. Similar to the arrangement of proteins in the lens, a crystalline arrangement of large proteins in the sarcomeres results in the formation of the contractile system, which is key to the essential function of cardiac myocytes. This system of contractile proteins is held in place, attached to the sarcolemma and the extracellular matrix via integrins in a non-contractile protein assembly known as costameres. Unlike simpler myofibrils in other cell types, striated muscle cells (cardiac and skeletal myocytes) have organized bundles of myofibrils associated with a reticulum of both modified smooth endoplasmic reticulum [sarcoplasmic reticulum (SR)] and mitochondria. While the former coordinate the excitation-contraction coupling from the sarcoplasmic depolarization via local release of calcium, the latter are responsible for production of high-energy phosphates to power the contraction. As myocytes are attached to each other with an intercalated disk consisting of proteins like actinin and vinculin, contraction of individual myocytes in series results in shortening of myofibrils; and simultaneous shortening of myofibrils in parallel drives myocyte contraction. Therefore, the sum of individual sarcomere shortening, both in series across individual myofibrils, as well as in parallel across myofibrils in the same bundle and across bundles in an individual myocyte, results in contraction of the entire myocyte and consequently the muscle.

Each sarcomere is bound by sarcomeric a-actinin-rich and electron-dense Z-lines on either side that form the essential platform as well as a central M-line that forms the two ends of the platform on which sarcomeric shortening occurs (Figure 1). Contractile proteins arranged as isotropic thin filaments attached to the Z-lines on either side are pulled inwards by the movement of anisotropic thick filaments attached to the central M-line, resulting in sarcomeric shortening. Simultaneous activation of the entire myofibril driven by coordinated calcium fluxes in the SR results in a summed contraction across the entire myofibril as well as myofibril bundles, and consequently contraction of the entire cell. While the arrangement of the motor proteins, actin and myosin in the thin and thick filaments respectively, results in contraction, it is the creation of a platform for these elements as well the anchors to the myofibril and cell architecture that are key to successful generation of a force from a functional sarcomere. Of these platform proteins, the giant proteins, titin, obscurin and nebulette, form the underlying structure on which the cardiac sarcomere is constructed. The largest protein in mammals, titin (MW 3.7 Md) (Tskhovrebova and Trinick, 2003), consists of both elastic and inelastic elements that are key to both structural and mechano-transductive functions. In addition, the titin skeleton provides a platform for both regulatory and degradative elements for repair of sarcomere elements and adaptation to stress.

As touched on previously, anchor proteins such as desmin, are essential to anchor the filaments to the Z-line as well as to costameres linking the myofibrils to the sarcolemma, the extracellular matrix as well as other myocytes via the intercalated (I) disk. In addition, desmin appears to be key in binding the mitochondria, the endoplasmic reticulum and the lysosomes to the myofibrils. Unlike mitochondria in other tissues, the reticular arrangement of these sarcomere-associated mitochondria is facilitated by desmin and result in enhanced functioning of these mitochondria (Milner et al., 2000). Thus, loss of desmin, during stress states results in both disruption of the sarcomere as well as mitochondrial dysfunction reflected in abnormal giant mitochondrial with swelling of the cristae and membrane depolarization (Milner et al., 1999; Maloyan et al., 2005; Diokmetzidou et al., 2016). Loss of desmin function, either due to mutation (Dalakas et al., 2000) or stress related post-translational modifications (Rainer et al., 2018; Tsikitis et al., 2018) result in destabilization of the sarcomere characterized by widening and loss of definition of the otherwise sharp electron dense actinin rich Z-lines and I-disks. Contractile dysfunction in this setting results from the disarray of the elements of the sarcomere as well as disruption of the attachment of the myofibrils to the cell membrane and ECM, as well as from the interaction with the nuclear lamina resulting in loss of nuclear homeostasis (Heffler et al., 2020). While initially identified in desmin-related genetic cardiomyopathies (Goldfarb and Dalakas, 2009), these features of desmin mis-localization, as well as sarcomeric disarray, are increasingly identified as features of cardiomyopathy in general (Coats et al., 2018; Tsikitis et al., 2018; Nakano et al., 2019).

While the contraction of an individual sarcomere is a remarkable marvel of electromechanical and metabolic coordination, the real achievement is the scaling of this in a coordinated syncytial pattern to the level of the myofibril, myocyte, and, ultimately, the cardiac muscle resulting in a functional heartbeat. Furthermore, this repeats, without fail, from the embryonic contraction of cardiac precursors through to the adult heart for many decades. In fact, until recently, the cessation of heartbeat was the *sine qua non* of death, in general, prior to recognition of “brain death.” With the exception of tonic contractility (albeit at lower frequencies) of the skeletal musculature powering respiration, cardiac myocyte function as well as the incredibly complex electromechanical cardiac syncytium is characterized by the amazing mechanical advantage generated by this unique morphology of the heart. This manifests with translating a 5–10% myofibril shortening into a 50–70% reduction in cardiac volume with each heartbeat. Thus, small disruptions in the contractility of individual sarcomeres result in equally dramatic and, potentially lethal, consequences to the organism. The close relationship of the sarcoplasmic reticulum, as well as sarcomere-associated mitochondria and lysosomes result in the ability to maintain unique metabolic signatures as well as a remarkable ability of the heart to endure during states of duress to the organism. Furthermore, the presence of direct points of communication (via integrins to the cell membrane and the extracellular matrix as well the between the myofibrils and the nucleus), allow the cardiac myocyte to maintain morphologic stability while dynamically adapting to continually variable hemodynamic, electromechanical, metabolic, and energetic changes (Henderson et al., 2017).

## Sarcostat: *A Proposed Framework to Understand Sarcomeric PQC*

**FIGURE 2:**
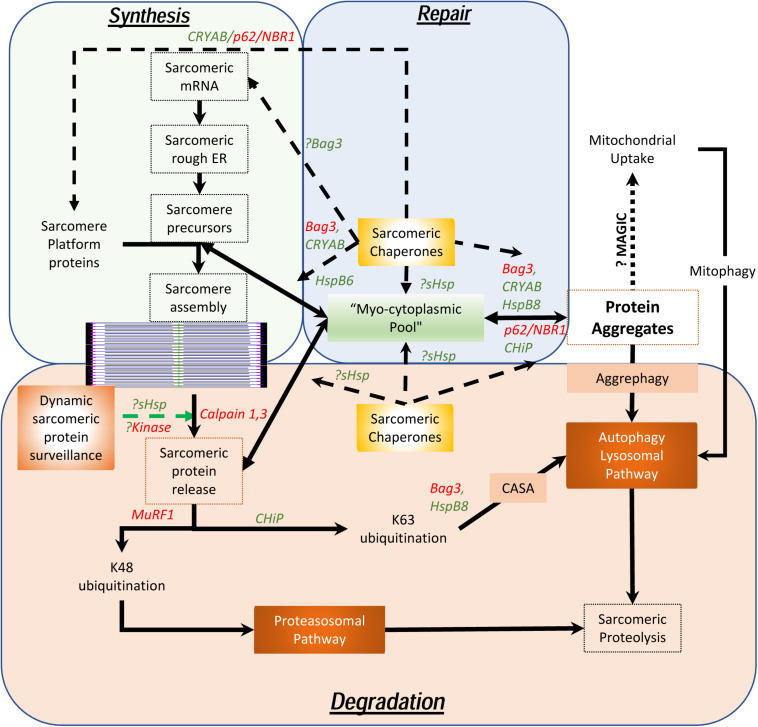
Proposed model of the cardiac sarcostat. In this model, mRNA and proteins are located in stoichiometric excess, in vicinity of the sarcomere, as a myocytoplasmic pool. As most sarcomeric proteins are highly insoluble and aggregate prone, concerted action of a variety of sarcomeric chaperones (in green letters) and chaperone associated sarcomeric proteins (in red letters) is required to ensure normal sarcomere structure and function. These chaperones also appear to be integral to sarcomere assembly as well as repair and maintenance of sarcomeric proteins, *in situ*. When the damaged proteins are detected (often due to exposure of kinase sites) by the “sarcostat damage sensing mechanism,” the proteins are removed from the sarcomere (via sarcomeric calpains) and returned to the myocytoplasmic reserve pool. When sarcomeric proteins acquire unfolded states due to nascent status, or are misfolded as a result of synthetic errors or due to damage, sarcostat chaperones assist in their refolding and in enabling dynamic equilibrium between the sarcomere-associated and the reserve pools. The proteins that are beyond the capacity of the refolding mechanism or require to be stored in the anticipation of continued stress, undergo aggregation under the action of Bag3, CryAB, and p62. As a result of liquid-liquid phase transition, these proteins and their cognate chaperones remain outside the osmotic load of the cell, stacked in a compact arrangement due to their intrinsic disordered domains (IDRs). These aggregates can be a source to release protein back in to the myocytoplasmic pool and, thus, the sarcomere. Normal lysosomal function and, possibly mitochondrial aggregate uptake (via a pathway termed as “MAGIC,” see text), result in continuous flux of these aggregates. In addition, normal proteolysis of isolated proteins occurs via the ubiquitin proteasome system and the autophagy-lysosomal pathway. During states when the ubiquitin proteasome pathway is inhibited, proteins are diverted into storage as protein aggregates or for degradation via the autophagy-lysosomal pathway. As many disease states also result in lysosomal dysfunction, the accumulation of the protein aggregates accompanies failure of the sarcostat. In these states, upregulation of the sarcostat protein degradation machinery (both UPS and ALP), results in improved sarcomeric protein quality, while concurrently increasing aggregate removal.

As maintenance of this assembly of large, otherwise insoluble, proteins is the key to homeostasis in chordates who depend on the continuous contractile function of the cardiac system, it is necessary to understand how these sarcomeres assemble, endure stress, remodel and how these proteins are degraded. Unlike the cardiac myocytes that have a remarkably long life and low (if any) replacement potential in post-natal life, myofibrils, costameres, and organelles have a comparatively short existence. Half-lives of sarcomeric proteins vary but most proteins are replaced within a matter of days and weeks (Martin, 1981; Rudolph et al., 2019). While hemodynamic stress results in increased protein synthesis (Schreiber et al., 1981), this is often accompanied by accelerated degradation, consistent with stable stoichiometry and steady state half-lives. Furthermore, it was recently demonstrated that both overexpression experiments with troponin I as well as with photobleaching experiments in a titin-GFP transgenic mouse line, that the overall stoichiometry suggests dynamic movement between the sarcomere and a reserve pool in the cytosol exists (Feng et al., 2009; da et al., 2011). This is remarkable given the fact that these proteins are regarded as highly insoluble and in the case of titin, remarkably large. Despite data showing that mRNA localizes to the sarcomere with local ribosomal synthesis of protein (Lewis et al., 2018), the question remains as to how cardiac myocytes are able to efficiently maintain the structure and function of the sarcomeres with accurate replacement of both damaged components and reintegrate “repaired” proteins, particularly in the setting of ischemic stress. To understand the dynamic nature of these processes, we propose the concept of a “sarcostat” (see Figure 2).

In addition to contractile and cytoskeletal elements, each sarcomere has a variety of proteins which function to maintain optimal contractility of the sarcomere. These include association of calpain 3 and 4 with the M-line; and of the autophagic adaptors, p62 and NBR1, on the titin kinase domain on the thin filament. Also, a host of specific ring finger proteins (MuRFs) are associated with the sarcomere, which function both as E3 ligases to facilitate protein degradation via K48 ubiquitination of sarcomeric proteins (Kedar et al., 2004) and for lysosomal targeting (or activation of signaling) via K63 ubiquitination (as occurs via CHiP) (Ulbricht et al., 2013). In contrast to their expected function, the sarcomeric calpains do not degrade proteins but merely facilitate removal of individual proteins from the sarcomere by making single cuts (Williams et al., 1999). In this proposed model (see Figure 2), the released proteins are then directed to the sarcostat (*infra vide*) and either recycled or degraded, via either the ubiquitin-protesomal system or the autophagy-lysosomal pathway. As many of the sarcomeric proteins are large complex structures, they need to be chaperoned to prevent misfolding and aggregation. For instance, the anchor protein desmin, which is essential for maintaining sarcomere integrity as well as cardiac sarcomeric mitochondria, is characterized by a large intrinsically disordered (low complexity) domain with a predisposition to form stable misfolded oligomers in the absence of its cognate chaperone αB-crystallin (Wang et al., 2001; Sharma et al., 2017; Kedia et al., 2019). This phenomenon is also seen in the case of filamin C, actin and myosin (Wojtowicz et al., 2015; Szikora et al., 2020). The discovery of variants of the giant protein titin in genomic studies of cardiomyopathy (Tharp et al., 2019), has also had a major impact in our understanding of how sarcomeric proteins are chaperoned in homeostasis and under stress. αB-crystallin appears to interact with the n2b region of titin in cardiac muscle and this binding plays a role in the stiffness of the sarcomere (Bullard et al., 2004). Similar to the effect of preventing aggregation of desmin, the binding of CRYAB to titin prevents aggregation of the disordered aggregation prone PEVK domain in titin. This facilitates optimal titin folding and maintains sarcomere elasticity (Kotter et al., 2014).

Studies have demonstrated that all sarcomeric proteins are produced far in excess of the actual observed protein content in the sarcomere (Lewis et al., 2018), and a significant fraction of sarcomeric proteins is actively in flux to and from a presumed “pool” to the sarcomere. Most of these proteins are large (if not giant), intrinsically insoluble and thus, aggregate prone. Although protein aggregation has been demonstrated during stress states (i.e., hemodynamic and metabolic stress), microscopy of normally functioning sarcomeres do not show where this “lost” protein pool of insoluble proteins exists or even why this stoichiometric excess production and flux exists. The concept of a myocytoplasmic “sarcostat” helps explain these apparent inconsistencies (see Figure 2). The sarcostat consists of a complex of chaperone proteins (Bag3, Unc-45, Hsp90 as well as the sHsps [HspB1 and HspB5-8 (including CryAB)], adapter proteins (p62, nBR1, and atg8), and “sensor” protein kinases and phosphatases (PKA, PKC isoforms, mTORC1, p38 MAPK, calcineurin and the titin kinase domain of titin). These are also complexed with anchor proteins (e.g., desmin), structural proteins (e.g., titin and obscurin), RNA binding proteins, sarcomeric ribosomes, adjacent sarcoplasmic reticulum, intra-sarcomeric calpain proteases, proteasome-directed (MuRFs) and autophagy-lysosome (CHIP/STUB) directed ubiquitin ligases, protein aggregates, stress granules, sarcomeric mitochondria and last, but not least, the sarcomere-associated lysosomes. In this model, the sarcostat is critical in continuously building, repairing, remodeling and degrading the sarcomere, thus reinforcing the notion of a dynamic sarcomere that actively and rapidly responds to changes in loading and environmental cues, rather than the prior static model of a singular contractile apparatus. Evidence for this model, albeit fragmented, already exists. In addition to the stoichiometric argument, the energetic argument to be able to draw on a local pool of protein, rather than solely relying on newly synthesized protein adds tremendous flexibility during states of metabolic stress when transcription and translation may be affected. Furthermore, augmentation of the protein quality control machinery (both proteasomal as well as the autophagy-lysosomal pathway) is associated with benefits and sarcomere recovery in a variety of heart failure models associated with sarcomere dysfunction even in the absence of correction of the primary genetic abnormality (such as mutations in MYH7, desmin, and CryAB) (Li et al., 2011; Pattison et al., 2011; Ranek et al., 2013; Gupta et al., 2014; McLendon et al., 2014; Cabet et al., 2015; Su et al., 2015; Dahl-Halvarsson et al., 2018; Ma X. et al., 2019). Thus, various elements of the “sarcostat” are in dynamic equilibrium, whereby perturbations in one element (either genetic or environmental) induce structural and functional abnormalities, and therapeutic targeting of this inciting stimulus or another balancing node can restore homeostasis.

## Protein Aggregation in Cardiac Myocytes: *Friend or Foe?*

A fundamental question in protein aggregate pathologies across multiple organ systems is whether protein aggregates are “good” or “bad” (Cox et al., 2018). When viewed as part of the static sarcomere model, protein aggregates have always been regarded as a pathologic, potentially toxic entity (Henning and Brundel, 2017). Indeed, akin to the pathology observed in neurodegenerative diseases, both pre-amyloid oligomers (Del Monte and Agnetti, 2014) and protein aggregates, consisting of a combination of normal sarcomeric proteins with or without mutated proteins, have been ascribed toxic roles. This notion is reinforced by studies that show that intracytoplasmic accumulation of these pre-amyloid oligomers, aggregates or their “toxic” constituents, recapitulates cardiotoxicity (Sanbe et al., 2004; Pattison et al., 2008, 2011); akin to a toxic role for pre-amyloid Aβ oligomers postulated as a pathogenic mechanism in Alzheimer’s disease (Demuro et al., 2011). Yet, the appearance of cardiac myocyte protein aggregates following hemodynamic stress in the pressure-overloaded left ventricle (Tannous et al., 2008) may be akin to the transient non-toxic Aβ plaques seen in traumatic brain injury (Scott et al., 2016) or reversible hyaline change in hepatic injury models (Kucukoglu et al., 2014); and may represent an adaptive state, to park large insoluble (and often ubiquitinated), proteins in a transitional state.

Understanding the dichotomous roles ascribed to such protein aggregates and assemblies will require experimental interrogation of their physico-chemical state using state of the art tools. A key feature of many protein aggregates is the presence of β-pleated sheets, which allow for efficient stacking of proteins as well as the concept of the liquid-liquid phase separation. Rather than conceptualizing these as precipitated solids in an otherwise liquid cytosol, protein aggregates can be considered as membrane-less organelles, similar to nucleoli, ribosomes, stress granules and P-bodies (Mitrea and Kriwacki, 2016; Uversky, 2017). As discussed in the subsequent section, protein aggregates in lower species are understood to play clearly adaptive roles as well as have potential toxic effects. The difference between the two functional states appears to be driven by the constituents of the aggregates rather than the aggregates themselves. Based on their currently understood role in protein aggregate formation in a variety of systems and species, the co-chaperone Bag3 (Meriin et al., 2018), the adaptor protein p62 (Komatsu et al., 2007; Sun et al., 2020) and small heat shock proteins (Ungelenk et al., 2016; Mogk and Bukau, 2017) (including the remarkably cardiac myocyte enriched chaperone αB-crystallin; Rajasekaran et al., 2007) appear to be critical in facilitating cardiac myocyte proteostasis. Of these, αB-crystallin (and its homologs) appears to be a universal component of these aggregates from bacteria to man (reviewed below). The presence of intrinsically disordered domains in all three of these proteins (Rauch et al., 2017; Wang et al., 2018; Haslbeck et al., 2019) as well as the potential for prion-like effects of proteins such as αB-crystallin that can exported via exosomes (D’Agostino et al., 2019) may result in both paracrine as well as potential endocrine effects.

In lower organisms, p62-enriched aggregates are believed to protect cells by sequestering toxic proteins [e.g., Keap1 (Pan et al., 2016) and mutant αB-crystallin (Zheng et al., 2011)]. In mammals, the preponderance of evidence points to protein aggregates being associated with cardiac pathology, suggesting that aggregates may be pathogenic (*vide infra*). Contrary to this assertion, the appearance of protein aggregates in cardiac myocytes after hemodynamic stress (pressure overload) in the myocardium (Tannous et al., 2008) may be adaptive as suggested by studies targeting TRIM21, a RING finger domain-containing ubiquitin E3 ligase that ubiquitylates p62 on lysine 7 to prevent is ability to aggregate (Pan et al., 2016). Mice lacking TRIM21 demonstrated near complete protection against pressure overload-induced left ventricular dilation and dysfunction, associated with marked aggregation of p62 and ubiquitylated proteins, suggesting that the inability to form protein aggregates worsens cardiomyopathy in this setting. This suggests that there is a “cinderella-zone” with respect to protein aggregate formation, akin to models seen in lower species (as discussed below). In this context, we speculate that the poorly understood “semi-crystalline” sarcomere assembly mechanism may share considerable similarity to the assembly of amyloid and protein aggregates; as mutations of key chaperones and components of the proposed cardiac sarcostat [i.e., Bag3 (Hishiya et al., 2010) and HspB7 (Mercer et al., 2018)] result in defects in sarcomere assembly, sarcomere maintenance and repair (see Figure 2).

A striking example of the physiologic role for such protein assemblies and aggregates was uncovered in studies focused on differentiation of neural stem and progenitor cells, wherein both ATP-dependent (TRiC/CCT) and ATP-independent sHsps (specifically CRYAB/HSPB5) promoted sequestration of mis-folded proteins into protective aggregates termed the “proteostat” to confer stress resilience (Vonk et al., 2020). Furthermore, the ability to form these protective aggregates declines with aging which may predispose to accelerated neurodegeneration with aging (Vonk et al., 2020); a premise that will require experimental testing in future studies. Another example of this phenomenon where heat shock proteins play a role in a “crystalline” structure is the role of HspA1 and HspB5 (αA- and αB-crystallin) in the lens (Horwitz, 2000). These findings support the notion that protein aggregates may not only be associated with pathology; but also play a protective role or trigger pathology in a context-dependent fashion.

While much evidence has been uncovered to understand the mechanisms for toxicity of aggregate prone proteins, such as the R120G mutant of αB-crystallin, potential mechanisms whereby protein aggregates confer cytotoxicity have largely remained unclear. In recent studies, we have uncovered a potential mechanism whereby protein aggregates induce toxicity in cardiac myocytes (Ma X. et al., 2019). Toxic mutations in aggregate-prone proteins (such as R120G αB-crystallin mutant) result in sticky aggregates that remove useful proteins (such as desmin) beyond the ability of the cardiac myocyte to compensate and result in sarcomere disruption and mitochondrial dysfunction as seen with expression of the R120G mutation in αB-crystallin that results in a desmin-deficient state (Ma X. et al., 2019). Moreover, some mutant proteins such as the R120G mutant of αB-crystallin result in very large and sticky amorphous hydrophobic aggregates (unlike those resulting from stacking of β-sheets) that not only remove useful proteins beyond the ability of the cardiac myocyte to compensate but also cause formation of mechanical intracellular barriers (Hipp et al., 2014; Mogk et al., 2018), and result in sarcomere disruption, and mitochondrial dysfunction. While cardiac myocytes attempt to correct this by upregulating other chaperones, as well as increasing activation of protein quality control pathways, namely the ubiquitin-proteasome system and autophagy; emerging evidence points to dysfunction in the ubiquitin-proteasome pathway at an earlier stage of the disease (Chen et al., 2005) and for autophagy-lysosome pathway dysfunction at late stages as a mechanism for disease progression (Ma X. et al., 2019). Indeed, serial assessment of the autophagy-lysosome pathway in a mouse model of R120G αB-crystallin-induced cardiomyopathy demonstrates early induction of autophagic flux with development of cardiac hypertrophy, followed by subsequent impairment with disease progression predating cardiomyopathic dysfunction (Pan et al., 2019). Mechanistically, this appears to secondary to mTOR activation likely secondary to long standing lysosomal amino acid release due to accelerated protein breakdown, which results in phosphorylation of TFEB (transcription factor EB, a master regulator of autophagy and lysosome biogenesis) and its inactivation with sequestration away from the nucleus on lysosomes and in the cytosol (Ma X. et al., 2019; Pan et al., 2019). Activation of the autophagy-lysosome pathway by intermittent fasting or targeted activation of transcription factor EB (Settembre et al., 2011) even at an advanced stage of disease pathogenesis was sufficient to restore normal function and rescue cardiomyopathy by restoring normal desmin localization (Ma X. et al., 2019; Mukai et al., 2019).

These observations suggest that a strategy targeting removal of aggregate-prone proteins may be effective in preventing or delaying cardiac pathology. Indeed, driving removal of aggregates via stimulation of the ubiquitin-proteasome pathway (Ranek et al., 2013; Gupta et al., 2014; Zhang et al., 2019) or of the autophagy-lysosome pathway (with activation of ATG7-stimulated autophagy or exercise; Bhuiyan et al., 2013) prevents toxicity of the R120G αB-crystallin mutant protein (Pattison et al., 2011; Pan et al., 2017) to attenuate cardiomyopathy development in this model. Another example of toxic protein aggregates and aggregate-prone desmin was uncovered in studies with modeling the cardiomyopathy-causing mutation H222P in the lamin A/C gene (Galata et al., 2018). Both a strategy of overexpressing αB-crystallin that resulted in chaperoning desmin to its physiologic location, or inducing haplo-insufficiency of desmin rescued cardiomyopathy by preventing desmin-induced sequestration of sarcomeric proteins from their physiologic location.

Taken together, there data suggest that aggregate-prone proteins, rather than protein aggregates are the initial drivers of pathology and their sticky nature makes protein aggregates “pathogenic” by hijacking and sequestering normal proteins at advanced stage of disease pathogenesis. Moreover, it is critical to recognize that all instances of proteostatic dysfunction do not manifest with aggregate pathology. Indeed, mutations in BAG3, a critical proteostatic mediator in cardiac myocytes induce cardiomyopathy without formation of protein aggregates, likely because BAG3 is required for aggregate formation (Fang et al., 2019). For example, studies modeling cardiomyopathy-associated mutations in BAG3 in iPSC-derived cardiac myocyte demonstrate myofibrillar disarray and marked proteostatic dysfunction without appearance of protein aggregates (Judge et al., 2017; McDermott-Roe et al., 2019). And, targeted ablation of BAG3 in the murine heart or expression of cardiomyopathy-associated BAG3 mutants induces myofibrillar degeneration (Hishiya et al., 2010) with increased portioning of proteins to detergent insoluble fraction (revealing their aggregate prone state) without formation of protein aggregates in the context of fulminant cardiomyopathic manifestations (Fang et al., 2017). While BAG3 plays a critical role in chaperone-assisted selective autophagy of proteins whereby its loss-of-function affects proteostasis (Ulbricht et al., 2013), BAG3 mutations are also associated with dysfunction of the macro-autophagy-lysosome pathway (Schanzer et al., 2018), which further impairs protein quality control mechanisms. Indeed, in instances where BAG3 mutations do induce protein aggregates and provoke cardiomyopathy, the mutant BAG3 protein acquires a gain-of-function aggregate-prone state, which forms protein aggregates with Hsp70, its natural binding partner and Hsp70 clients (Meister-Broekema et al., 2018). These data points to a critical need for mechanisms to efficiently remove damaged and dysfunctional proteins as an effective countermeasure against development of pathology.

These data suggest that the sarcomere functions in a semiautonomous state of proteostasis with independent components for protein synthesis (peri-sarcomeric ribosomal complexes and sarcomeric mRNA), and sarcomere-linked chaperone proteins (sHsps and Hsp90 analogs as well as p62 and Bag3) that facilitate folding of key sarcomeric proteins (see Figure 2). These components also appear to play a role in stabilizing and maintaining the “reserve” sarcomeric protein to provide a ready source of replacement parts to ensure continuous function. Furthermore, sarcomere damage due to stretch and load, ischemia, and heat stress result in misfolding of components. These “damaged” components are released from the sarcomere by calpains and enter the “reserve” pool where the cognate chaperones assess the integrity of the protein and either assign these for removal via the uniquitin-proteasome system, or via the autophagy lysosomal pathway. In the latter, this takes the form of either chaperone-assisted selective autophagy (Tan and Wong, 2017) mediated by Bag3, p62, CHIP, and sHsps (HspB8 and CryAB) or via aggrephagy of protein aggregates directly. Each of these elements is proposed to contribute to the proposed cardiac sarcostat (Figure 2). Thus, failure of the sarcostat is predicted to engender sarcomere disruption and contractile dysfunction, culminating in cardiomyopathy, heart failure and death.

## Heat Shock Proteins: *With a Little Help From My Chaperones*

As discussed in the prior sections, cardiac myocytes are unusually large cells that are dependent on complex quaternary structures of protein complexes to maintain homeostasis (Figure 1). Thus, it is critical to understand the biology of the chaperones that helps maintain an appropriate folded state of these proteins from synthesis, through deployment, and finally, to removal and degradation. Initially seen in *Archaea* as small moieties conferring resistance against heat denaturing insults (Macario et al., 1991), the so-called heat-shock proteins have evolved into a multitude of classes and, remarkably, have retained their underlying structure and function through the course of evolution.

Heat shock proteins can be broadly classified as large and small heat shock proteins. Large heat shock proteins (70–90 kDa) are known to have ATPase function and use energy dependent mechanisms to fold (foldase) proteins (Moran Luengo et al., 2019). By contrast, small heat shock proteins (15–30 kDa) have traditionally been thought to be energy-independent chaperones that sequester proteins and prevent misfolding (i.e., holdase) (Janowska et al., 2019). Newer data (as discussed subsequently) indicate that their function, both in isolation as well as in concert with large Hsps and other co-chaperones may be more complex, and sHsps may function in both BAG3-dependent and independent manner (reviewed in Fang et al., 2019).

Another class of heat shock proteins exist in bacterial, fungal and plant systems, i.e., the Hsp110 AAA + ATPase disaggregases that can disassemble amyloid and protein aggregates (Torrente and Shorter, 2013). Recent studies indicate that proteins with disaggregase function (some with Hsp homology) exist in the animal kingdom but their role appears to be unclear (Baker et al., 2017; Taguchi et al., 2019; Avellaneda et al., 2020). In this context, it is notable that physiologic or reversible protein aggregates are observed in yeast as a reserve pool of proteins to respond to stress (Saad et al., 2017). These amyloid proteins are disrupted by a yeast protein disaggregase, hsp104, which has been lost in metazoans and can drive rapid ATP and Hsp70/40-dependent disaggregation of amyloid protein in both yeast and metazoan cell types (Yokom et al., 2016; Gates et al., 2017; Shorter, 2017). Indeed, Hsp104, when exogenously introduced into models of neurodegenerative diseases, namely Parkinson’s disease and frontotemporal dementia has demonstrated efficacy in disaggregating TDP-43, FUS, and α-synuclein with resulting attenuation of cellular pathology (DeSantis et al., 2012; Jackrel et al., 2014). Whether these protein systems are functional in mammalian cardiac myocytes or can be harnessed for therapeutic potential, remains to be explored.

Another exciting recent discovery has been the observation that mitochondria participate in taking up cytosolic misfolded proteins to facilitate their aggregation on the mitochondrial surface via a mitochondria-mediated proteostasis mechanism, termed MAGIC (mitochondria as guardian in cytosol; see Figure 2; Ruan et al., 2017). These aggregates are subsequently removed by mitochondrial fission and subsequent mitophagy to remove the fissioned-off mitochondria (Li et al., 2019). Hsp104 can forcibly disaggregate these mitochondrial protein aggregates and target their import into the mitochondrial matrix for degradation by Pim1 (LON protease). Whether mitochondrial handling of cytosolic protein aggregates participates in cardiac myocytes homeostasis and stress response, remains unknown.

A unifying factor across all heat shock proteins is HSF-1, the master regulator of the heat shock response in eukaryotes (Gomez-Pastor et al., 2018); which was demonstrated to be essential for thermos-tolerance in mammalian systems using a targeted genetic approach (McMillan et al., 1998). The HSF family of transcription factors (HSF1-6 in humans) appears to not only drive the various heat shock proteins but also induce a concerted array of stress response genes that respond to a variety of stimuli, including heat, oxidative stress, metals and proteotoxicity (Murshid et al., 2018). In response to stress, inactive monomeric HSF-1 is activated resulting in formation of a DNA-binding homotrimer via leucine-zipper domains. This multimerization results in activation of the bipartite NLS and nuclear translocation where the DNA binding N-terminal helix-turn-helix domain binds to the nGAAn consensus sequence on promoters. A bevy of heat-shock proteins (as well as 14-3-3, VCP, and TRiC proteins) are able to hold the HSFs in a monomeric state and are part of a feedback loop to prevent continued activation of the HSF target gene activation (Gomez-Pastor et al., 2018).

In mammals, Hsp70 and Hsp90 are the most prominent class of the large Hsp family of proteins. Both of these are notable for the presence of a nucleotide-binding domains, peptide-binding domains and variable C-terminal regions (reviewed in Moran Luengo et al., 2019). The Hsp90 proteins have greater substrate specificity as compared with the Hsp70 family. By and large, Hsp70 proteins play a role in protein folding from the nascent polypeptide chains at the ribosomes, through complex quaternary structures prior to protein deployment. In contrast, Hsp90 proteins collaborate with C-terminal Hsp-Interacting Protein (CHIP) and BAG3, directing their actions to specific targets (Ranek et al., 2018). Both of these proteins are key elements of the intracellular “sarcostat” in cardiac myocytes (Figure 2), and have been observed to play important roles in the pathogenesis of heart failure. Interestingly, in the setting of αB-crystallin R120G mutation, while overexpression of the foldase Hsp70 is unable to rescue the phenotype resulting from misfolded unchaperoned desmin, while overexpression of the holdase sHsps (HspB5, 6, and 8) was sufficient to confer rescue (Hussein et al., 2015). This suggests that many of the properties of the individual classes of proteins are more nuanced *in vivo* as compared to *in vitro* predictions.

Working hand-in-hand with these energy-dependent chaperones, small Hsps consist of a relatively diverse family of proteins with molecular weights mostly ranging from 15 kDa through 40 kDa. Unlike the large ATP-dependent Hsps that are conserved through fungi and eukaryotes, mammalian sHsps are also conserved with those in prokaryotes, *Archae* as well as viruses. Previously believed to be mere “holdases” that are critical in holding proteins in stable conformations in the cytosol, new evidence indicates far more diverse and complex roles (Fang et al., 2019; Haslbeck et al., 2019; Janowska et al., 2019). The sHsps (HspB1-10 in humans) are present in many tissues, and observed to play roles in nearly every disease from infections through degenerative diseases. Of these, HspB-1, 2, 3, 5, 6, 7, and 8 have been shown to be present at relatively high levels in the heart with significant functional roles noted in both mouse models as well as human disease. With the exception of HspB7, which has a significant monomeric function, all of these appear to function as chaperones in an oligomeric state. This varies from dimers and trimers in the case of Hspb6 and HspB8 (Bukach et al., 2004; Shatov et al., 2018) to the 30–40-mers seen with CryAB (HspB5) (Aquilina et al., 2003; Janowska et al., 2019). Nonetheless, each of these sHsps and all of their evolutionary forebearers, are characterized by the presence of a β-sheet enriched “α-crystallin” domain (ACD) (Horwitz, 1992; Janowska et al., 2019) consisting of 6–8 β-sheets. Flanking this are relatively disordered N- and C-terminal (NTR and CTR) regions. While the CTR is rich in polar amino acids and may play a role in solubility (Janowska et al., 2019), the NTR is hydrophobic and may play a role predominantly in substrate specificity. Despite the overall structural similarities, the sHsps vary in presence of I/VXI/V motifs in either the CTR or the NTR that result in interaction of either regions with the hydrophobic cleft of the ACD. Notably, the CTR interacts with the hydrophobic groove in ACD between (β4 and β8) (Janowska et al., 2019). These folding events in sHsps appear to be key in determining their chaperone function as well as the multimerization, either as homo-polymers or heteromers.

## CRYAB and Shsps Through Evolution: *The Long and Winding Road*

Cardiac myocytes are unique with regards to structure, function and replacement potential. Similar to prokaryotes and yeast, survival of individual cardiac myocytes is critical to maintaining cardiac architecture and function. Thus, it is likely that many mechanisms that are essential for monad survival may also be specifically relevant to cardiac myocyte homeostasis, but not necessarily for homeostasis in other replicating cell types. Given the importance of proteostasis in cardiac myocytes (as discussed above), looking for phylogenetic survival and proteostatic pathways in lower organisms could be key in understanding the role of human cardiac proteostasis in homeostasis. Of the multitude of proteins that participate in the proteostatic pathways, αB-crystallin/CRYAB//HspB5 is unique in being very heavily expressed in the cardiac myocytes (3–5%) of total cardiac protein (Bennardini et al., 1992). At baseline, αB-crystallin functions as a chaperone as a ∼24–40 mer, with a soccer ball shaped 0.5–1 mDa complex (Aquilina et al., 2003). Stress-induced activation of p38 MAPK (Ito et al., 2001) results in phosphorylation of CryAB at S59 (Simon et al., 2007), thus changing it from a 24 to 32-mer to a 6-mer and is associated with increased partitioning to the insoluble fraction. This phenomenon is observed in the myocardium during ischemia-reperfusion (Golenhofen et al., 1998), oxidative stress (Prasad et al., 2013), hyperglycemia (Reddy et al., 2014), high fat diet (Prasad et al., 2013), hemodynamic stress with transverse aortic constriction (Pereira et al., 2014), and chronic heart failure (Dohke et al., 2006; Marunouchi et al., 2013; Fung et al., 2017); and appears to portend a poorer prognosis in human studies (Clements et al., 2007, 2011). From a functional standpoint as a chaperone, 30–40-mer multimeric αB-crystallin chaperone binds the N2B subunit of titin (Bullard et al., 2004), thus preventing unfolding and colocalizes with the Z-line, along with desmin (Ma X. et al., 2019). This association is disrupted by the R120G mutation, associated with Z-line disruption, as seen in heart failure models (Zhu et al., 2009). While the αB-crystallin/HspB2 double knockout (due to overlapping exons), has increased stress induced cardiomyopathy with ischemia-reperfusion injury (Morrison et al., 2004) and myocardial pressure overload (Kumarapeli et al., 2008), the phenotype is not seen with a functional HspB2 knockout (Ishiwata et al., 2012), reinforcing αB-crystallin’s importance. As aging is often associated with protein aggregates and increased αB-crystallin S59 phosphorylation, it is interesting that the αB-crystallin/HspB2 null is protected against ischemia in aging mice (Benjamin et al., 2007). Therefore, understanding and extrapolating the properties and phenomena associated with primordial homologs of this unique cardiac enriched protein hold considerable promise for development of targeted therapeutics for myocardial pathology.

An interesting example of a bacterial crystallin homolog is Hsp16.3 in Mycobacterium tuberculosis. Functioning as a chaperone, this protein is able to facilitate the survival of the bacterium by promoting the dormant state during stress (Jee et al., 2018). Similarly, the chaperone sHsp16 in *Trypanosoma cruzi* functions by allowing the organism to resist oxidative and heat stress (Perez-Morales et al., 2009). However, the earliest example of sHsp is MjHsp16.5 in the archaean, *Methanococcus jannaschii* (Feil et al., 2001). Analysis of the ACD shows considerable homology with the MTB Hsp16.3, Ohhsp16.9 (rice), Hsp16.2 (*C. elegans*); as well as murine HspB6 and bovine CRYAB/HspB5 (Kim et al., 1998). The most impressive demonstration of the significance of sHsp homologs appears to be in *C. elegans* where the lifespan prolongation in the ultra-long-lived insulin resistant *daf-2* mutant was dependent on protein aggregates containing the CryAB homolog Hsp16.1 (Walther et al., 2015). Furthermore, recent work indicates that a non-canonical sHsp (Hsp-17) functions as an “aggregase” and loss of function mutants have shorter lifespan (Iburg et al., 2020). Similarly, the yeast analog Hsp42, harboring a prion-like domain in the N-terminus, is endowed with both chaperone and aggregase functions (Grousl et al., 2018). As in *C. elegans*, this aggregase function appears to be critical for proteostasis in heat stress (Grousl et al., 2018). In yeast, there are subcellular deposition sites called the “insoluble protein deposit (IPOD)”, where, upon exposure to environmental stress, damaged or misfolded proteins are targeted for degradation or refolding helped by molecular chaperones (Rothe et al., 2018). Soluble protein aggregates are targeted to JUNQ/INQ (juxtanuclear or intranuclear aggregates), or to the CytoQ (cytoplasmic accumulation); whereas amyloid aggregates accumulate in IPOD site (Rothe et al., 2018). In *Drosophila*, sHSPs have diverse functions. Hsp23, Hsp26, and Hsp27 could be involved in embryo morphogenesis by their ability to bind actin and microtubule (Goldstein and Gunawardena, 2000; Gong et al., 2004; Fisher et al., 2008; Hughes et al., 2008). Hsp26 has been shown to interact with myosin 10A, the *Drosophila* myosin XV homolog, a protein involved in regulating filopodial dynamics during dorsal closure (Liu et al., 2008). Hsp22 is the sHsp preferentially expressed during aging and its level of expression is partially predictive of longevity in individual flies (King and Tower, 1999; Yang and Tower, 2009). *Drosophila* host defense against pathogenic bacteria, fungi and viruses involves Toll, Imd, JNK, JAK-STAT, and p38 MAPK pathways (Eleftherianos and Castillo, 2012; Kingsolver et al., 2013); and these pathways activate Hsf and requires the proper expression of Hsp26, Hsp27, Hsp60D, and Hsp70Bc to mediate host defense (Chen et al., 2010). These observations point to the evolutionary conserved nature of sHsp biology as well as the remarkable ability of organisms to harness their potential to sustain critical life-sustaining processes, which culminate in mechanisms that maintain cellular homeostasis in highly specialized and long-lived cell types such as the cardiac myocytes.

## Targeting Heat Shock Proteins for Cardioprotection: *Let’s Come Together to a Better Place*

Understanding and exploring sHsps as a therapeutic target has been at the forefront of protein quality-centric efforts to prevent and treat pathology. Studies have demonstrated protective effects of exogenous sHsps on cardiac myocytes under various stresses, *in vitro*: (1) with expression of multiple heat shock proteins in ischemia (reviewed in Martin et al., 1997); (2) with activation of HspB1 in preventing aggregate formation with R120G αB-crystallin mutant expression (Zhang et al., 2010); and (3) with αB-Crystallin expression that prevents adrenergic stimulation-induced hypertrophic growth (Kumarapeli et al., 2008). Transgenic overexpression of αB-crystallin was effective in restoring mitochondrial quality and rescuing cardiac myocytes death in mice with genetic ablation of desmin (Diokmetzidou et al., 2016), a mouse model for desminopathies that result from loss of function of desmin due to genetic mutations. Cardiac myocyte targeted overexpression of αB-crystallin was also sufficient in attenuating development of dilated cardiomyopathy in a mouse model of H222P mutation in Lamin A/C gene, by restoring desmin localization (Galata et al., 2018). Transgenic αB-crystallin overexpression in cardiac myocytes protects against development of pathologic hypertrophy by attenuating NFAT activation after pressure overload (Kumarapeli et al., 2008); and αB-crystallin interacts with focal adhesion kinase and protects its proteolysis by calpains under stretch, protecting cardiac myocytes from apoptosis under pressure overload stress (Pereira et al., 2014). αB-crystallin was also shown to be a part of the cardiac sodium channel complex by interacting with Nv1.5, the pore-forming submit, with effects on increased sodium channel density and current (Huang et al., 2016); pointing to the potential for harnessing this biology toward treatment of arrhythmias induced by sodium channel dysfunction. Substantial evidence has also accumulated indicating a beneficial role for BAG3 gain of function in protecting against various stress stimuli. *In vitro* studies have demonstrated the efficacy of exogenous BAG3 in protecting against hypoxia-induced cell death, (Zhang et al., 2016), improving mitochondrial quality in hypoxia-reoxygenation injury (Cheung et al., 2019), in suppressing αB-crystallin R120G mutant-induced protein aggregation and cell death (Hishiya et al., 2011) and in nuclear protein quality control under proteotoxic stress (Gupta et al., 2019). Analogously, we have demonstrated that TFEB-induced upregulation of HspB8, a BAG3 partner, was essential for chaperoning desmin back to its physiologic localization state in a mouse model of R120G αB-crystallin induced cardiomyopathy (Ma X. et al., 2019). Our findings with shRNA mediated knockdown on HspB8 demonstrated that the benefits of enhancing the autophagy-lysosome-pathway on R120G-induced cardiomyopathy were lost with loss-of-function of HspB8. HspB8 (Hsp22) also plays a critical role in cardiac homeostasis as mice with germline ablation of HspB8 develop worse cardiomyopathy and increased mortality as compared with wild-type controls in response to pressure overload (Qiu et al., 2011). Interestingly, transgenic expression of BAG3 in cardiac myocytes reduced small heat shock protein levels specifically leading to a reduction in αB-crystallin and HspB1 accompanied by development of cardiomyopathy (Inomata et al., 2018), pointing to the critical stoichiometric balance with these protein families in cardiac physiology.

Targeted activation of the large heat-shock chaperone family members has also been explored as a potentially useful target for cardioprotection. Transgenic expression of Hsp70 or its interacting protein CHIP (Carboxyl terminus of Hsp70-interacting protein (CHIP), a ubiquitin ligase) was protective against doxorubicin-induced cardiomyopathy (Naka et al., 2014; Wang et al., 2016). Furthermore, an aggregate of studies suggest that activation of Hsp70 signaling in protective against cardiac ischemia-reperfusion injury (Song et al., 2019). However, a note of caution is relevant given a role for Hsp70 described in promoting cardiac hypertrophy in response to pressure overload, which is typically pathologic and results in decompensation (Kee et al., 2008). Also, treatment with a Hsp90 inhibitor attenuated activation of Ras/Mek/Erk mitogen activated protein kinase (MAPK) signaling pathway to attenuate cardiac hypertrophy in the remote non-infarcted myocardium in the post-myocardial infarction left ventricle (Tamura et al., 2019).

Intriguingly, recent studies point to the exciting prospect of employing oxysterols to alter the aggregation properties of cHsps such as the R120 mutant of αB-crystallin (Makley et al., 2015; Molnar et al., 2019), and cataract-causing Y118D mutant in αA-crystallin (Zhao et al., 2015) which were highly effective in restoring protein solubility in the lens to attenuate established cataracts. Understanding how heat shock proteins are regulated via post-translational mechanisms (Gomez-Pastor et al., 2018) will be essential to develop novel therapeutics (such as oxysterols) to therapeutically target them for prevention and treatment of cardiac pathologies.

## Conclusion

Cardiac myocytes are characterized by the roles of semi-crystalline protein assembly (the sarcomere) as well as by the various roles of the cardiac-enriched sarcostatic oligomeric complexes of heat shock proteins, i.e., the “crystallins.” These crystalline proteins mirror their function in the ocular lens, to turn large insoluble proteins into a dynamic robust and durable machine with uninterrupted function through the lifetime of an organism. While prior work indicated that akin to neurodegeneration, the appearance of protein aggregation was purely a pathogenic phenomenon, recent studies indicate that a more nuanced approach is necessary. An enhanced understanding of the evolutionarily preserved small heat shock proteins (that share the same oligomeric properties from *Archaea* to man), as well as the potential protective roles of amyloid and aggregates in lower species associated with these sHsps is essential in developing new sarcomere-preserving strategies. It is our hope that development of such sarcomere-targeted approaches will foster development of the next generation of therapies for heart failure.

## Author Contributions

All authors participated in drafting the manuscript and approved it prior to submission.

## Conflict of Interest

The authors declare that the research was conducted in the absence of any commercial or financial relationships that could be construed as a potential conflict of interest.
